# Supplementary material for: Potential for Zika virus transmission by mosquitoes in temperate climates
Source: Proc Biol Sci. 2020 Jul 8;287(1930):20200119. doi: 10.1098/rspb.2020.0119 (PMC7423484; doi:10.1098/rspb.2020.0119)
Supplement: ESM [file rspb20200119supp1.docx]

**Potential for Zika virus transmission by mosquitoes in temperate climates**

**Electronic Supplementary materials**

Article published in: Proceedings of the Royal Society B: Biological Sciences

DOI: 10.1098/rspb.2020.0119

**Authors**

Marcus S C Blagrove^1,2,*^, Cyril Caminade^1,2^, Peter J Diggle^3^, Edward I Patterson^4^, Ken Sherlock^1^, Gail E Chapman^1^, Jenny Hesson^1,6^, Soeren Metelmann^1,2^, Philip J McCall^5^, Gareth Lycett^5^, Jolyon Medlock^7^, Grant L Hughes^4^, Alessandra della Torre^8^, Matthew Baylis^1,2^

**Affiliations**

1: Department of Epidemiology and Population Health, Institute of Infection and Global Health, University of Liverpool, UK.

2: National Institute of Health Research Health Protection Research Unit in Emerging and Zoonotic Infections, University of Liverpool, UK.

3: Lancaster Medical School, University of Lancaster, Lancaster, UK.

4: Departments of Vector Biology and Tropical Disease Biology, Centre for Neglected Tropical Diseases, Liverpool School of Tropical Medicine, Liverpool, UK.

5: Vector Biology Department, Liverpool School of Tropical Medicine, Liverpool, UK.

6: Department of Medical Biochemistry and Microbiology, Zoonosis Science Center, Uppsala University, Sweden.

7: Medical Entomology and Zoonoses Ecology, Public Health England, HPA, Salisbury, UK.

8: Department of Public Health & Infectious Diseases, Sapienza University of Rome, Laboratory. Affiliated to Instituto Pasteur Italia – Fondazione Cenci Bolognetti, Rome, Italy.

**Corresponding author**

Marcus Blagrove. Liverpool Science Park - Innovation Centre 2, 131 Mount Pleasant, University of Liverpool, Liverpool, UK, L3 5TF. +447 964 660 678. ORCiD: 0000-0002-7510-167X. [marcus.blagrove@liverpool.ac.uk](mailto:marcus.blagrove@liverpool.ac.uk)

**Part A – Model Supplementary Information**

***Derivation of R_0_(T) for ZIKV – Ae. albopictus model***

The basic reproduction number R_0_ for ZIKV (one vector – one host model) was derived from a one host, two vector R_0_ model [1]. Given recently published evidence, the model has been slightly modified. R_0_(T) is given by:

$$R_{0}(T)=\left( \frac{b\beta{a(T)}^{2}}{\mu(T)} \right)\left( \frac{\nu(T)}{\nu(T)+\mu(T)} \right)\left( \frac{m}{r} \right)$$

The biting (a), mortality (µ) and extrinsic incubation ($\nu$) rates depend dynamically on temperature (see Fig. S4 and table S1 for the related analytical functions). All other parameters are fixed to a constant value (see Table S1). The final R_0_ estimate was standardized to range between 0 and 1, to be consistent with previously published models and make our results directly comparable to such studies [2]. We used standardized R_0_ (by dividing by the maximum) which ranges between 0 and 1, consequently values of constant parameters do not affect the shape of the standardized R_0_(T) curve. This standardized R_0_ function is then used as a simple metric for the relative suitability of temperature for transmission, rather than an absolute metric to define secondary cases based on R_0_, following [2]. The R_0_ > 1 threshold highly relies on the vector to host ratio parameter (m) which is very heterogeneous, difficult to measure in the field (as it heavily relies on trap type) and to model [3]. We then focus on mean annual R_0_ values which exceeds important thresholds based on our laboratory experiments. Based on our experimental results we know that *Ae. albopictus* cannot transmit ZIKV at 17ºC, but some transmission might occur in between 17 and 19ºC. Above 19ºC, we know that ZIKV transmission by *Ae. albopictus* does occur, at least in a laboratory setting. Consequently, we highlight standardized R_0_ values ranging between 0.201 and 0.295 which respectively corresponds to T=17ºC-33.6ºC and T=19ºC-33.1ºC (Fig. S4d), and we define standardized R_0_(T) categories above that threshold [0.3-0.6; 0.6-0.8; 0.8 and above]. We then extrapolate our R_0_(T) curve to risk maps, using the same standardized R_0_ thresholds and colour code, using observed gridded temperature data for the recent period and climate model projections for the future. Further details about climate datasets are available in the following section “Observed climate datasets and climate change scenarios input data”. Observed presence points of *Ae. albopictus*, derived from [4] are overlaid onto standardized Ro maps for the recent period on Figure 2. We provide global risk maps and regional zooms over Europe, North America, South America, Asia and Africa.

For consistency with a former published study [5], we incorporate rainfall seasonality effects to mask desert regions. We utilized a rainfall criterion, derived for malaria in Africa within the Mapping Malaria Risk in Africa (MARA) project framework, e.g. “80 mm per month for at least five months for stable transmission” [5]. If this criterion was not met we assumed R_0_(T)=0 for a particular grid cell and month. We also switched off the MARA rainfall criterion to investigate the additional impact of rainfall on our temperature driven R_0_(T) model.

**Table S1: Standardized R_0_(T) model parameter settings**. *denotes parameters which are dynamically simulated in space and time over the whole time period. T stands for temperature.

| **Symbol** | **Description** | **Constant/Formula** | **Comments** | **Ref** |
| --- | --- | --- | --- | --- |
| *a(T) | Biting rate (per day) | a=(0.0043T + 0.0943)/2 | The linear dependency to temperature was based on estimates for *Ae. aegypti* in Thailand. Biting rates for *Ae. albopictus* were halved based on published observed feeding interval data | [6,7] |
| b | Transmission probability - vector to host (0-1) | b=0.5 | Baseline value of ref [8]. Note that constant parameter values should not impact the shape of the final standardized R_0_(T) estimate (std R_0_(T) was rescaled to range between 0-1, see Fig S3d) | [8] |
| β | Transmission probability - host to vector (0-1) | β=0.0665 | Baseline value of ref [8]Note that constant parameter values should not impact the shape of the final standardized R_0_(T) curve (std R_0_(T) was rescaled to range between 0-1, see Fig S3d) | [8] |
| *μ(T) | Mortality rate (0-1 per day) | μ=1/(1.1+exp(-4.04+0.576T))+ 0.11883 if T < 15°C  μ=0.000339T^2^-0.0189T+0.336 if 15°C ≤ T < 26.3°C  μ=1/(1.065+exp(32.2-0.92T))+ 0.073079 if T ≥ 26.3°C | Mortality rate was derived for both mosquito vectors from published estimates based on both laboratory and field data. Due to discontinuity around the different temperature thresholds these estimates have been updated. | [9] |
| *υ(T) | Extrinsic Incubation Rate (days) | 1/ υ = EIP10_albo = -1.0757T+43.0342  EIP10_detr = -1.07567T+46.025 | EIP10(T) was estimated based on our updated laboratory data (see Figure 1). EIP(T) for *Oc. detritus* is also shown for comparison. | This study |
| m | Vector to host ratios | m= 12.9 | Calculated as maximum vector to host density ratios (508/39.4] in ref [7] Note that constant parameter values should not impact the shape of the final standardized R_0_(T) curve (std R_0_(T) was rescaled to range between 0-1, see Fig S3d) | [8] |
| r | Recovery rate (per day) | r=1/7 | 1 week viraemia is a common value for ZIKV. | [10] |

***Derivation of the length of the ZIKV transmission season (LTS) – Ae. albopictus model***

To investigate seasonality in risk, we calculated the length of the transmission season (LTS) based on our standardized R_0_(T) estimates. If std R_0_(T) > 0.295 (corresponding to T=19ºC-33.1ºC e.g. orange and red colours on Fig. 2d) for a particular location and month, we assumed that temperature conditions were suitable for ZIKV transmission (so we assign 1 to particular location and month); conversely if std R_0_(T) =< 0.295, we assumed that no transmission occurred (so we assigned 0 to that particular location and month). We then sum months at risk on annual basis to derive LTS which ultimately ranges between 0-12 months.

***Observed climate datasets and climate change scenarios input data***

For the recent period, we used the GHCN-CAMS gridded temperature data which combines station data from the Global Historical Climatology Network (GHCN) version 2 with the Climate Anomaly Monitoring System (CAMS) [11]. This monthly temperature dataset is available at 0.5° x 0.5° spatial resolution at global scale for the period 1948-2019. For rainfall, we employed the Global Precipitation Climatology Centre (GPCC) global rainfall data available at the same spatial and time resolution for the same time period [12]. We calculated annual average for the 1980-2010 baseline period, as advised by the World Meteorological Office guidelines [13].

Calibrated General Circulation Model (GCM) temperature outputs from the ISI-MIP project were utilized [14] to assess future risk of ZIKV transmission. In this study, we used the RCP2.6, RCP4.5, RCP6.0 and RCP8.5 emission scenarios (from the lowest to the highest emission scenario) based on the ensemble mean of 5 GCMs (hadgem2-es, ipsl-cm5a-lr, miroc-esm-chem, gfdl-esm2m, noresm1-m). We conducted analysis for the future periods 2040-2059 (2050s – mid 21^st^ century changes) and 2070-2089 (2080s – late 21^st^ century changes).

***R code***

http://dx.doi.org/Id/dryad (non-working link, code are available in the Dryad data repository, under embargo pending acceptance)

**Part B – Experimental Data Supplementary Information**

**Tables S2: Relative titre of ZIKV in saliva of *Oc. detritus* (A) and *Ae. albopictus* (B)**. All titres are shown relative to the average of all titres (calculated using Ct values as measured by qRT-PCR, primer efficiency, and made relative to the average of all values here), this was done for simplicity of interpretation and because a known titre control was not used. Each cell shows: the average titre, the number of positive samples, and the standard deviation. Below each table is the overall average and standard deviation for all positive samples of the species. The difference between the two species is significant (P < 0.00001, Mann-Whitney U-Test, two tailed).

1. ***Oc. detritus***

|  | **Days post infection** | | | | | |
| --- | --- | --- | --- | --- | --- | --- |
| **Temperature** |  | **10** | **14** | **17** | **21** | **28** |
|  | **19°C** |  |  | 0.109  1  NA |  | 0.160  1  NA |
|  | **21°C** |  | 1.209  2  1.634 | 0.392  1  NA | 0.181  1  NA | 0.193  2  0.129 |
|  | **24°C** |  | 0.090  1  NA | 0.507  1  NA | 0.299  3  0.351 | 0.417  2  0.463 |
|  | **27°C** | 0.170  1  NA | 0.556  2  0.575 | 0.313  3  0.334 | 0.226  2  0.109 |  |
|  | **31°C** | 0.576  1  NA | 0.067  2  0.024 |  |  |  |

Average = 0.3599, Standard deviation = 0.4832

1. ***Ae. albopictus***

|  | **Days post infection** | | | | | | |
| --- | --- | --- | --- | --- | --- | --- | --- |
| **Temperature** |  | **7** | **10** | **14** | **17** | **21** | **28** |
|  | **19°C** |  |  |  | 0.655  1  NA | 1.951  1  NA | 2.015  2  2.392 |
|  | **21°C** |  | 0.181  1  NA |  | 0.507  1  NA | 0.863  3  0.950 | 1.410  2  1.808 |
|  | **24°C** |  | 0.614  1  NA | 0.394  2  0.431 | 1.192  2  1.256 | 0.475  1  NA | 1.639  4  1.703 |
|  | **27°C** |  | 0.699  1  NA | 0.460  2  0.164 | 2.689  1  NA | 1.380  3  1.283 | 1.707  4  1.047 |
|  | **31°C** | 0.090  1  NA | 1.167  1  NA | 0.472  2  0.147 | 1.432  4  1.689 | 1.338  2  0.695 | 2.842  5  3.363 |

Average = 1.3541, Standard deviation = 1.5382

**Table S3: Number of mosquitoes in each condition for Figure 1 and Supplementary Figure 1.** Number of mosquitoes tested in each condition. The number for transmission rows show the total number of saliva samples tested from surviving individuals (either positive or negative) (note that all saliva positive mosquitoes were also body positive); the number for survival shows the total mosquitoes (either dead or alive) at the time-point. Note that the number of mosquitoes used on each day was not equal, given experimental constraints on the numbers of mosquitoes that can be infected at one time, fewer mosquitoes were used on less informative time points (0 and 5 days) compared to later time points.

|  |  |  | **Day** | | | | | | | |
| --- | --- | --- | --- | --- | --- | --- | --- | --- | --- | --- |
| **Species** | **Condition** | **Temperature (°C)** | **0** | **5** | **7** | **10** | **14** | **17** | **21** | **28** |
| ***Oc. detritus*** | **Transmission** | **17** | 3 | 5 | 10 | 10 | 14 | 15 | 17 | 20 |
|  |  | **19** | 3 | 5 | 7 | 11 | 12 | 19 | 16 | 18 |
|  |  | **21** | 3 | 5 | 6 | 10 | 20 | 15 | 16 | 16 |
|  |  | **24** | 3 | 4 | 7 | 9 | 17 | 21 | 16 | 17 |
|  |  | **27** | 3 | 5 | 8 | 10 | 21 | 17 | 15 | 0 |
|  |  | **31** | 3 | 5 | 8 | 9 | 13 | 0 | 0 | 0 |
|  | **Survival** | **17** | 3 | 6 | 10 | 11 | 15 | 19 | 20 | 23 |
|  |  | **19** | 3 | 5 | 7 | 12 | 14 | 21 | 19 | 20 |
|  |  | **21** | 3 | 5 | 7 | 13 | 21 | 19 | 21 | 20 |
|  |  | **24** | 3 | 4 | 7 | 12 | 18 | 24 | 21 | 24 |
|  |  | **27** | 3 | 6 | 9 | 13 | 31 | 24 | 27 | 34 |
|  |  | **31** | 3 | 9 | 14 | 35 | 47 | 43 | 50 | 47 |
| ***Ae. albopictus*** | **Transmission** | **17** | 5 | 10 | 10 | 14 | 11 | 15 | 12 | 12 |
|  |  | **19** | 5 | 10 | 10 | 13 | 11 | 15 | 13 | 14 |
|  |  | **21** | 5 | 10 | 10 | 16 | 11 | 16 | 15 | 14 |
|  |  | **24** | 5 | 10 | 10 | 13 | 15 | 17 | 13 | 16 |
|  |  | **27** | 5 | 10 | 10 | 14 | 13 | 16 | 15 | 16 |
|  |  | **31** | 5 | 10 | 11 | 16 | 11 | 19 | 14 | 17 |
|  | **Survival** | **17** | 5 | 10 | 10 | 15 | 13 | 19 | 16 | 15 |
|  |  | **19** | 5 | 10 | 10 | 14 | 14 | 18 | 15 | 17 |
|  |  | **21** | 5 | 10 | 11 | 17 | 13 | 19 | 20 | 18 |
|  |  | **24** | 5 | 10 | 10 | 15 | 18 | 22 | 17 | 22 |
|  |  | **27** | 5 | 11 | 11 | 17 | 18 | 20 | 20 | 24 |
|  |  | **31** | 5 | 11 | 15 | 22 | 17 | 25 | 25 | 35 |


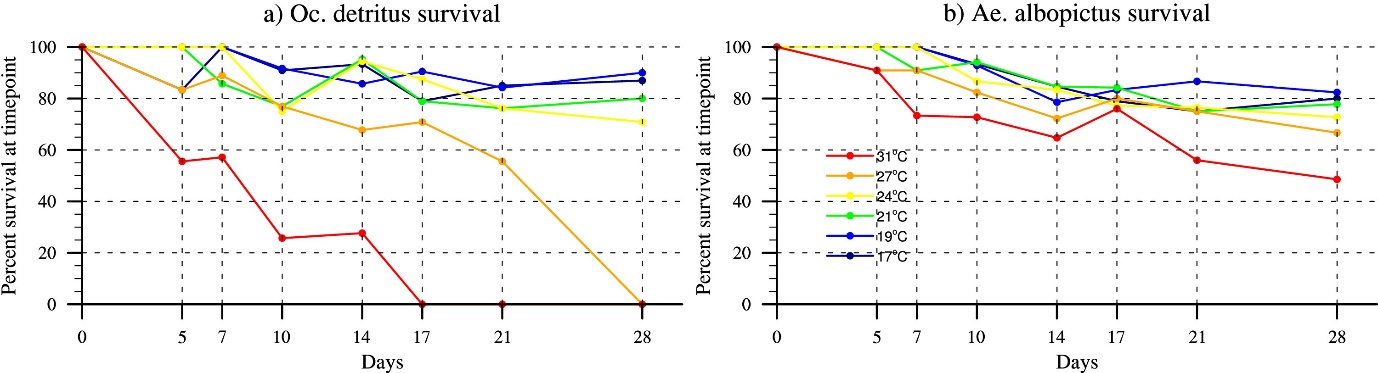


**Figure S1: Mortality of *Oc. detritus* and *Ae. albopictus***. The survival of *Oc. detritus* and *Ae. albopictus* was compared over six different temperatures (17°C, 19°C, 21°C, 24°C, 27°C and 31°C) and eight time points (0, 5, 7, 10, 14, 17, 21, and 28 days post infection). The number of mosquito individuals at each condition is shown in tables S2. (Note that survival is measured from an independent cohort for each time point, hence may ‘increase’ over time if a later cohort had higher survival).

**Small-scale experiments on *Cx. pipiens pipiens* and *Cs. annulata*.**

We found no evidence that field-obtained *Cx. pipiens pipiens* or *Cs. annulata* were competent for ZIKV after 17 days maintenance at 21°C (Table S4). However, only relatively small numbers of these two species were tested due to practical limitations (extremely low feeding rate for *Cx. pipiens pipiens* and difficulty in collecting large numbers of wild larvae for *Cs. annulata*). Because of the small numbers, the individuals were tested at 21°C only; this temperature was chosen to provide a balance between excessive mortality at high temperatures and extremely long EIPs at lower temperatures (which leads to higher mortality prior to the EIP being reached). Because of the low numbers, we could not conclude that these species are not competent for ZIKV.

**Table S4 – Mortality and competence of field-obtained *Cx. pipiens pipiens* and *Cs. annulata*.**

| Species | Number fed | Day 17 mortality (%) | Day 17 ZIKV positive |
| --- | --- | --- | --- |
| *Cx. pipiens pipiens* | 52 | 15 (28.8) | 0 |
| *Cu. annulata* | 24 | 8 (33.3) | 0 |

**Table S5: Results of CPE assay**. Grey cells indicate CPE was observed. (dpi = days post infection)

| **Sample** | **Cytopathic effect (CPE)? Yes(Y)/No(N)** | | | | | | |
| --- | --- | --- | --- | --- | --- | --- | --- |
|  | 1 dpi | 2 dpi | 3 dpi | 4 dpi | 5 dpi | 6 dpi | 7 dpi |
| Positive control | N | Y | Y | Y | Y | Y | Y |
| Negative control | N | N | N | N | N | N | N |
| 1 | N | N | Y | Y | Y | Y | Y |
| 2 | N | N | N | N | N | N | N |
| 3 | N | N | N | N | N | N | N |
| 4 | N | Y | Y | Y | Y | Y | Y |
| 5 | N | N | N | N | N | N | N |
| 6 | N | N | N | N | N | N | N |
| 7 | N | Y | Y | Y | Y | Y | Y |
| 8 | N | N | Y | Y | Y | Y | Y |
| 9 | N | N | N | N | N | N | N |
| 10 | N | N | N | N | N | N | N |


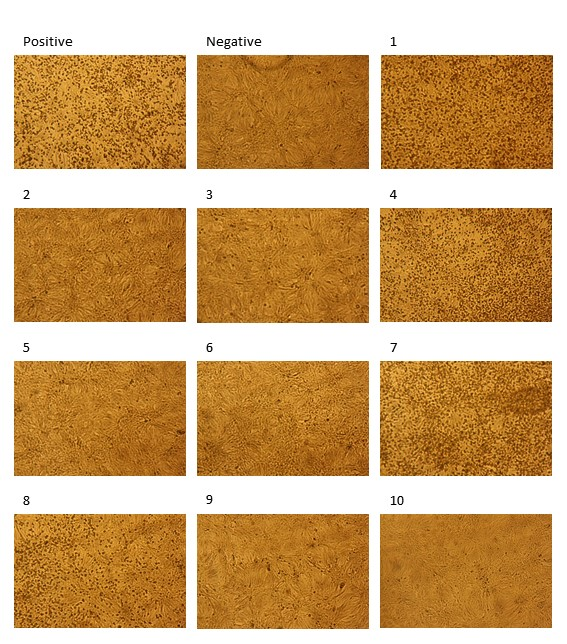


**Figure S2: CPE assay photographs**. Pictures of CPE assay shown in table S5 from day 4. Positive and negative controls shown, with all 10 pools of three saliva samples.

**Part C – Additional Analyses and Maps.**

***Models of extrinsic incubation period (EIP_10_)***

Our models were limited to data for 19 to 31^o^C as no positives (saliva or body) were detected at 17^o^C.

An unknown proportion of mosquitoes is assumed to be susceptible to infection; the remainder (the refractory proportion) do not become saliva-positive, even under optimal conditions. The aim was to determine, at each time point (*d*) and temperature (*t*), the proportion, p_s_, (where s = 1 for *Ae. albopictus* or 2 for *Oc. detritus,* that were saliva-positive for ZIKV out of the unknown susceptible proportion, Model 1 thereinafter).

Our model for p_s_ (t,d) is an extended logistic model, with a plateau effect representing the proportion of mosquitoes susceptible to saliva infection, estimated from the maximum proportion with bodies positive for ZIKV. Based on exploratory analysis of whole-body infection proportions, we assume that for each species the plateau value (δs) is reached by day 21 and does not depend on temperature (over 19°C). The basic model form is:

$$\log\left( \frac{p_{s}(t,d)}{\delta s-p_{s}(t,d)} \right)= \alpha+\beta t+\delta d$$

and that at 21 days and 28 days, P(body-positive) = δs.

As a possible simplification of the aforementioned model, we considered replacing the two plateau values δs: s = 1,2, by a single parameter, δ (Model 2). As a possible elaboration of the model, we considered allowing temperature (Model 3) and/or day effects (Model 4) to be non- linear. We also tested interactions between all dependent variables (species, time and temperature) in Models 6, 7 and 8.

We compared all models by generalized likelihood ratio tests. To test for non-linearity of temperature or day effects, we compared Model 1 with a model in which temperature or day was treated as a factor on 5 or 8 levels, respectively. For our final model we estimated parameters by maximum likelihood, and calculated confidence intervals by applying the asymptotic multivariate Normal sampling distribution of the maximum likelihood estimators; for the δs parameters, we carried out the calculations on the log-odds scale, log{δs/(1 − δs)}. One property of the fitted model that is of particular interest is EIP_10_, the average time to 10% positivity. As this is a non-linear function of the model parameters, we calculate estimates and confidence intervals for EIP_10_ by Monte Carlo sampling from the multivariate Normal sampling distribution of the maximum likelihood parameter estimates.

Because the plateau effect takes the model outside the class of generalized linear models, we wrote our own functions in R^TM^ version 3.5 [15] to fit the model (see Sx text for the code).

Table S6 gives the results of generalized likelihood ratio tests of various models. We conclude that the preferred model is Model 1, with different plateaus for each species, as it fits the data significantly better than Model 2 with a single plateau for the two species. Model 1 is not significantly different from Models 3-7.

Table S7 shows maximum likelihood estimates and 95% likelihood-based confidence intervals for the parameters in model (1).

Supplementary figure 1 shows survival rates for *Oc. Detritus* (Fig S1a) and *Ae. albopictus* (Fig S1b) while outputs from Model 1 and our experimental data are shown on fig. S3 and table S7.

**Table S6**: Models, maximised log-likelihoods (Lmax), numbers of parameters (m), comparisons (null vs alternative), generalized likelihood ratio statistics (deviance, D) and p-values (p).

| Model | *L*max | *m* | Comparison | *D* | *p* |
| --- | --- | --- | --- | --- | --- |
| 1  equation (1) | -203.47 | 5 |  |  |  |
| 2  single plateau (δs = δ) | -205.40 | 4 | 1 vs 2 | 3.8570 | 0.0495 |
| 3  temperature non-linear | -202.34 | 3 | 1 vs 3 | 2.2644 | 0.5194 |
| 4  time non-linear | -199.88 | 11 | 1 vs 4 | 7.1877 | 0.3038 |
| 5  species-by-temperature interaction | -203.37 | 6 | 1 vs 5 | 0.2099 | 0.6468 |
| 6  species-by-time interaction | -203.46 | 6 | 1 vs 6 | 0.0166 | 0.8969 |
| 7  temperature-by-time interaction | -202.55 | 6 | 1 vs 7 | 1.8409 | 0.1748 |

**Table S7**: Maximum likelihood estimates and 95% confidence intervals of parameters in model 1.

| Parameter | Estimate | Lower 95% CI | Upper 95% CI |
| --- | --- | --- | --- |
| δ1 | \| 0.2741 \| \| --- \| | 0.2155 | 0.3418 |
| δ2 | 0.2003 | 0.1452 | 0.2698 |
| α | -9.1690 | -13.2127 | -5.1253 |
| β | 0.2134 | 0.0842 | 0.3427 |
| γ | 0.2060 | 0.1019 | 0.3102 |


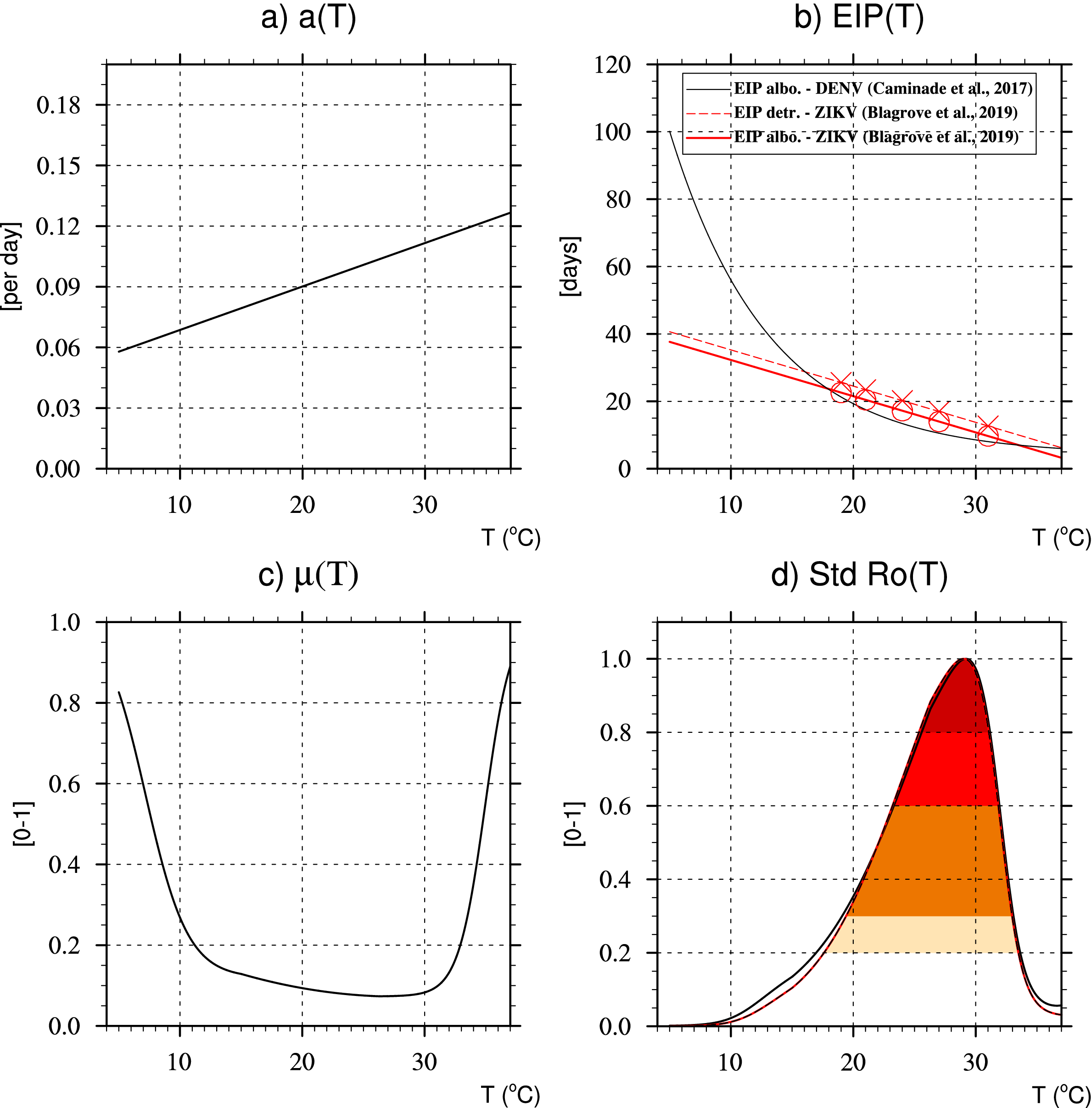


**Fig. S3 –Extrinsic Incubation Periods (EIPs, in days) of ZIKV for *Ae. albopictus* and *Oc. detritus* complemented by standardized R_0_(T) estimates for *Ae. albopictus* potential to transmit ZIKV.** a) Biting rate (per day) dependency on temperature for *Ae. albopictus* [6,7]*,* b) extrinsic incubation period of ZIKV infection (in days) for both *Ae. albopictus* and *Oc. detritus*, the stars and open circles depict simulated EIP_10_ values (Table 1) at experimental temperature points for *Oc. detritus* and *Ae. albopictus* respectively, c) Mortality rate (µ) for *Ae. albopictus* [9], d) Standardized Basic reproduction number (R_0_ – average number of new cases one case generates, in an otherwise fully susceptible population) dependency on temperature for *Ae. albopictus* (red line); a former published estimate for *Ae. albopictus* and dengue virus (DENV) is depicted by the black solid line [16]. Standardized R_0_(T) values above 0 are highlighted in different colours; the beige colour depicts standardized R_0_(T) values [ranging between 0.201 and 0.295] for which *Ae. albopictus* might become infected by ZIKV in the laboratory [17-19ºC]; orange, red and dark red colours depict temperatures above 19ºC (ZIKV transmission by *Ae. albopictus* occurs in the laboratory above that temperature threshold, see Fig. 1a).


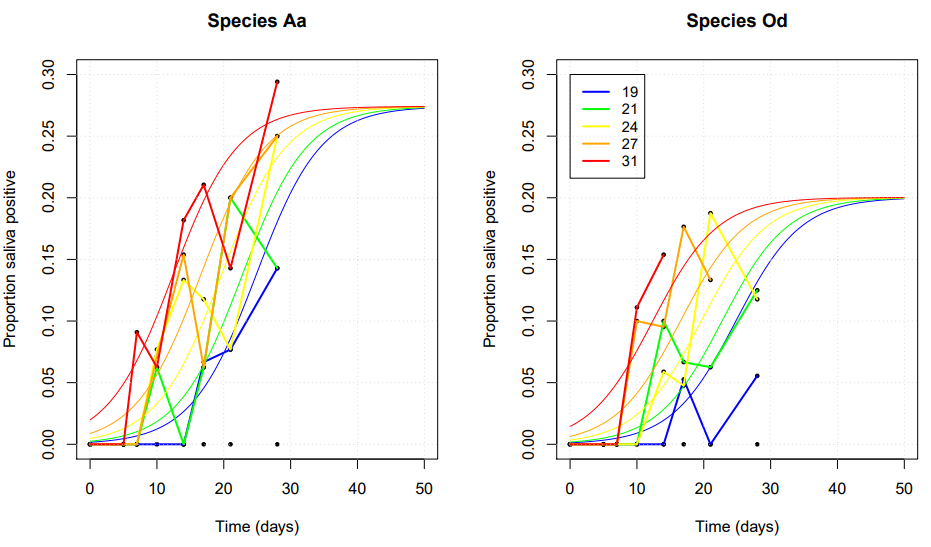


**Figure S4: Logistic regression models of ZIKV infection in *Oc. detritus* and *Ae. albopictus***. Points are the proportion of mosquitoes saliva-positive for ZIKV after incubation at temperatures from 17-31°C, for the shown number of days, out of those susceptible to infection; the susceptible proportion is set by the upper limit of the body infections. Points have been horizontally jittered to prevent overlay. Straight lines join sequential points, curved lines are the fitted logistic models for each species (*Oc. detritus* and *Ae. albopictus*).


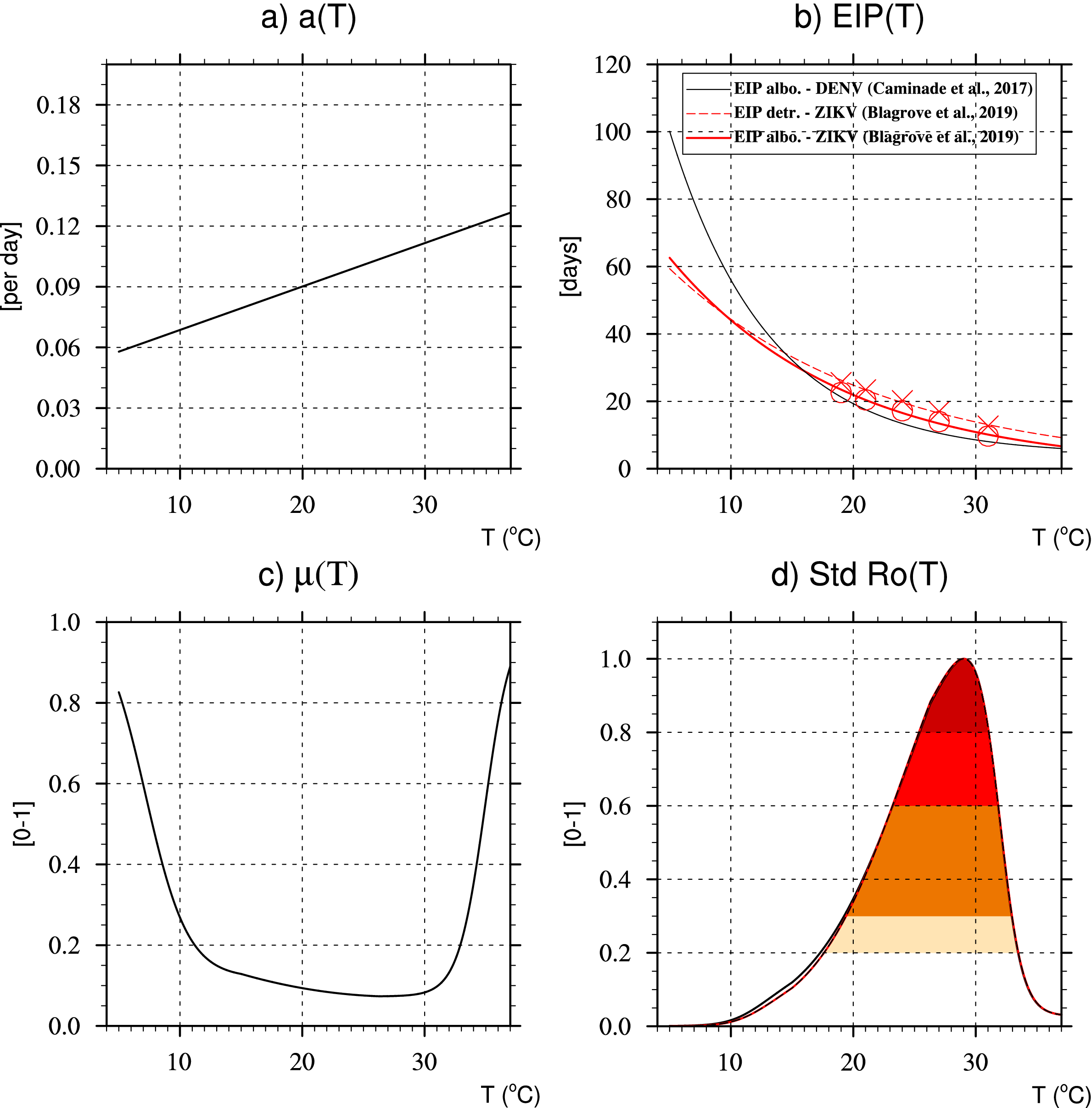


**Figure S5 – Extrinsic Incubation Periods (EIPs) of ZIKV for *Oc. detritus* and *Ae. albopictus complemented by R_0_(T) estimates for Ae. albopictus potential to transmit ZIKV (same as Fig. S3, but we now use an exponential instead of linear fit for EIP10 on FigS3b)*.** a) Biting rate dependency to temperature for *Ae. albopictus* [6,7]*,* b) extrinsic incubation period of ZIKV infection for both *Ae. albopictus* (eip_albo(T) = 88.8505exp(-0.0700339 * T) and *Oc. detritus* (eip_detr(T)=79.4215exp(-0.0581906T)), derived from exponential fitting; T denotes temperature. the stars and open circles depict simulated EIP_10_ values (shown in Table 1) at experimental temperature points for *Oc. detritus* and *Ae. albopictus* respectively. c) Mortality rate (µ) for *Ae. albopictus* [9], d) Standardized R_0_(T) values above 0 are highlighted in different colours; the beige colour depicts standardized R_0_(T) values [ranging between 0.201 and 0.295] for which *Ae. albopictus* might become infected by ZIKV in the laboratory [17-19ºC]; orange, red and dark red colours depict temperatures above 19ºC (ZIKV transmission by *Ae. albopictus* occurs in the laboratory above that temperature threshold, see Fig. 1a).


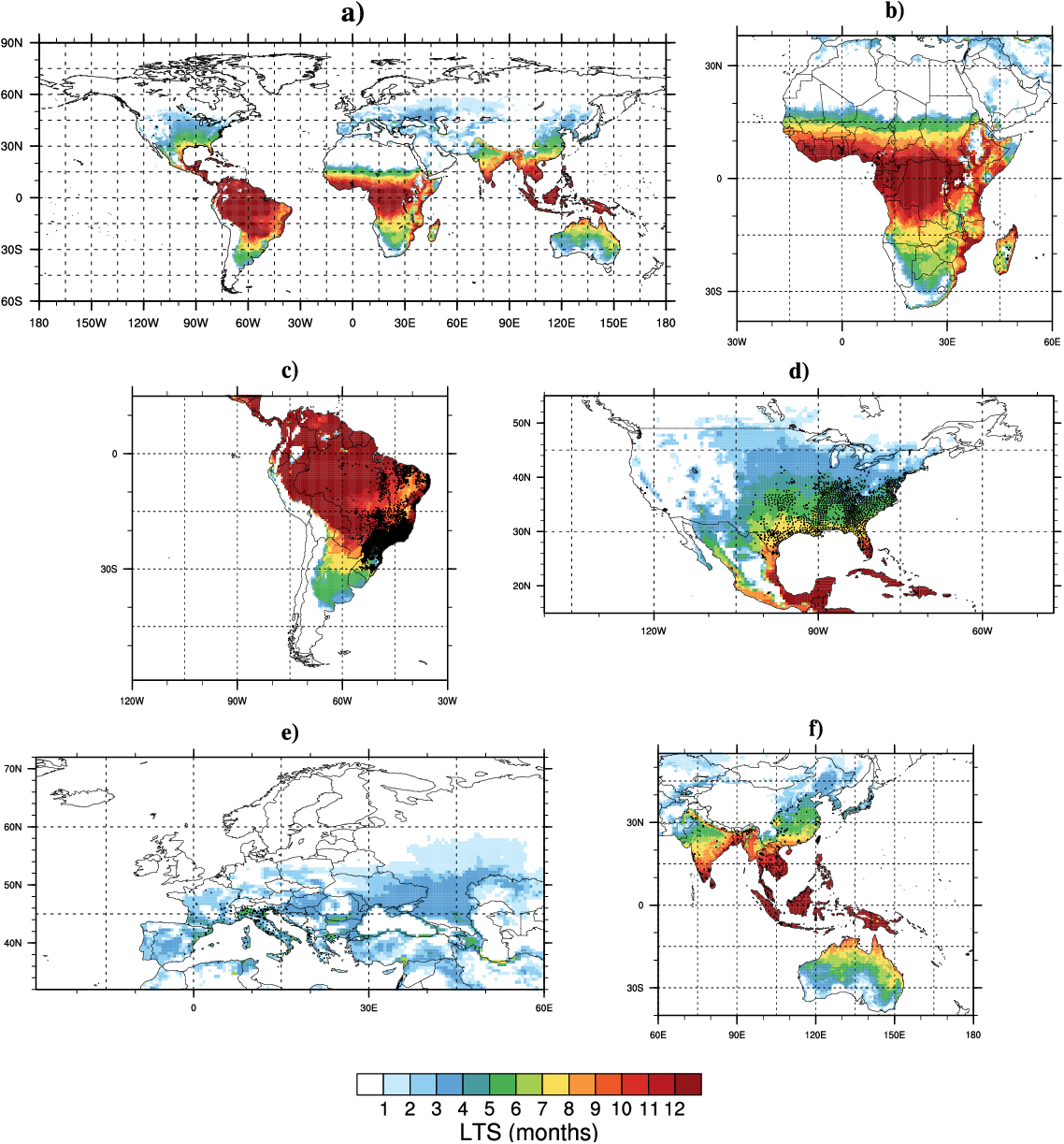


**Figure S6**: Simulated length of the ZIKV transmission season (LTS in months) based on observed rainfall and temperature data (1980-2010) for a) the globe, b) Africa, c) South America, d) North America, e) Europe and f) Asia.


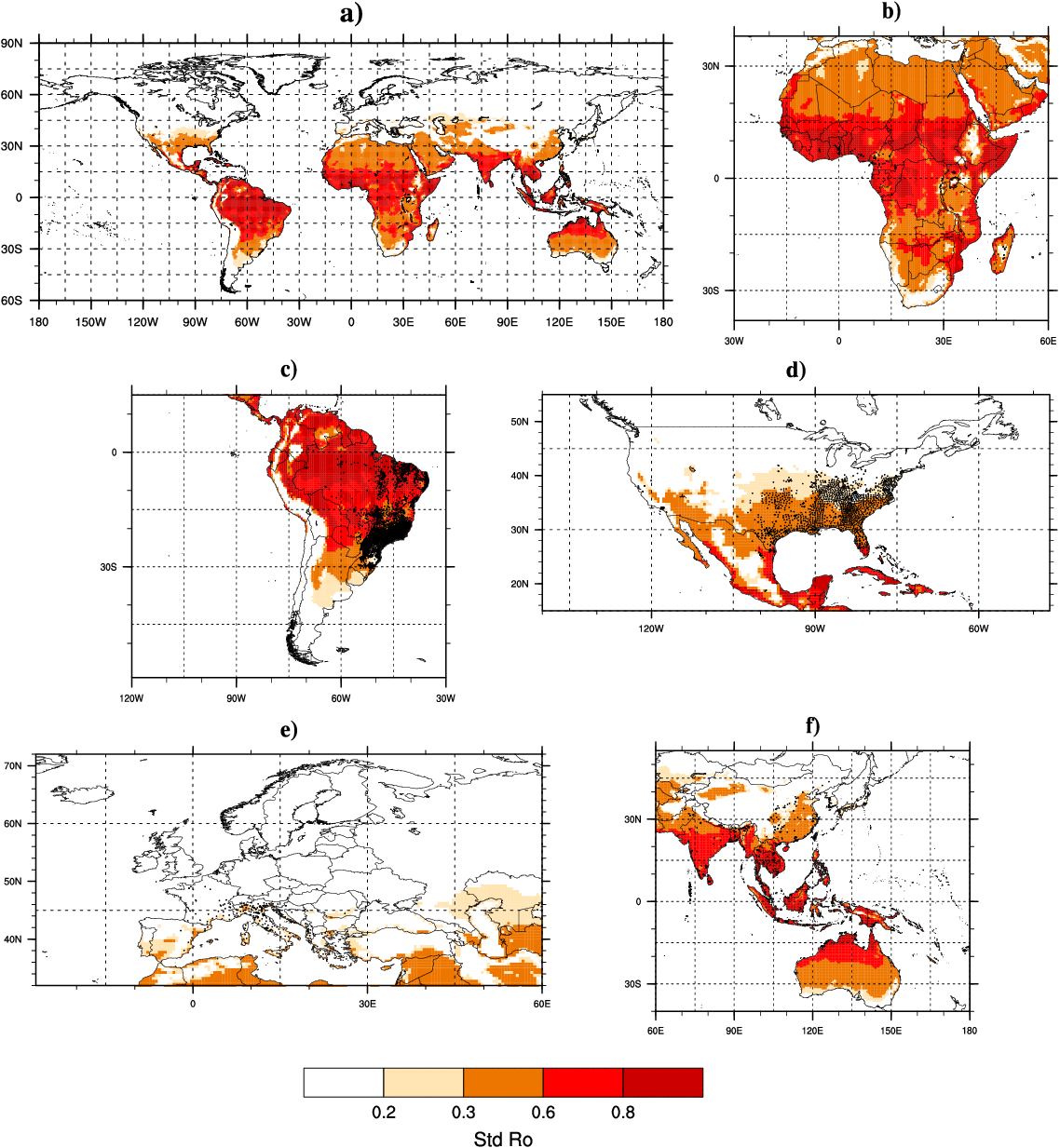


**Figure S7:** same as figure 3 in the main text - but the MARA rainfall criterion has been switched off (temperature effect only).


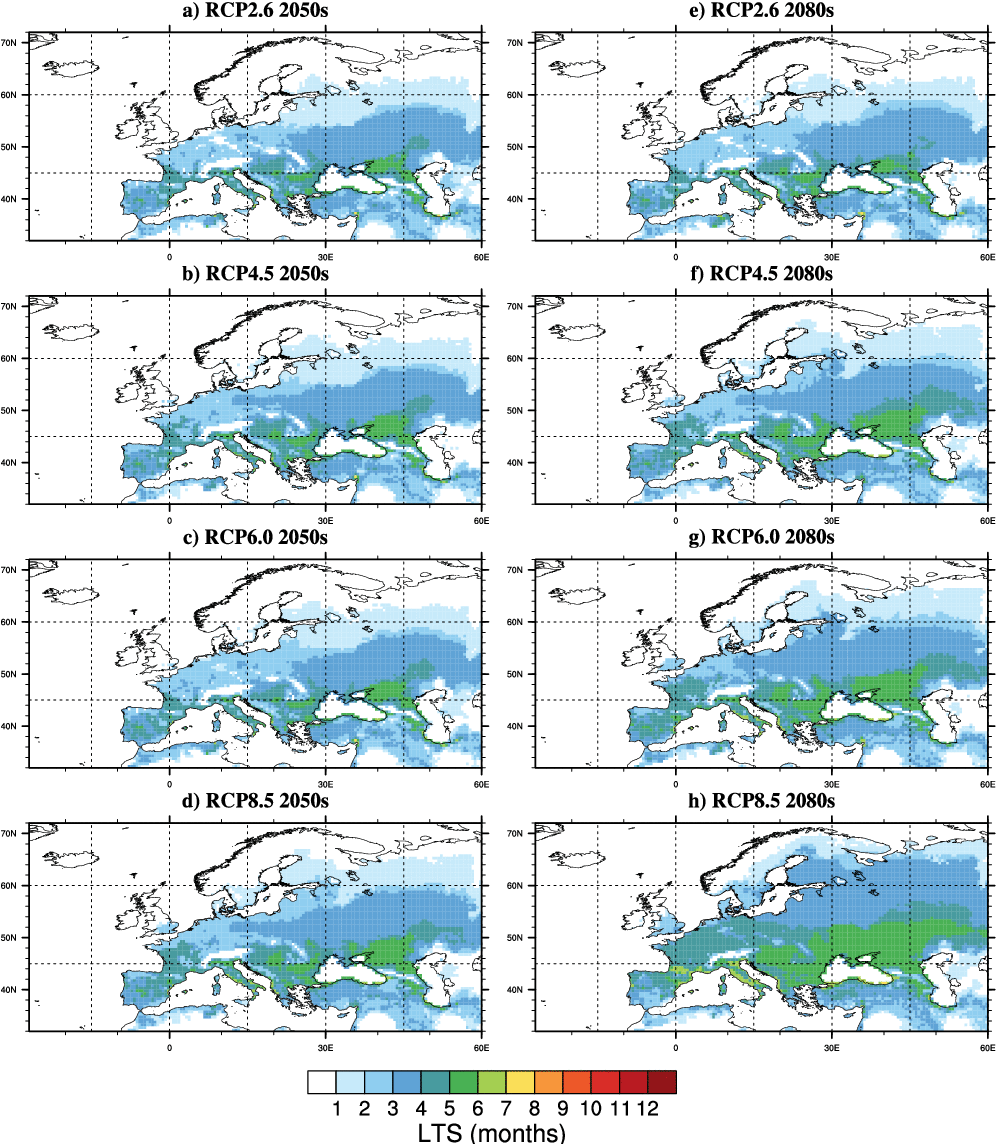


**Figure S8**: **Simulated length of the ZIKV transmission season by *Ae. albopictus* over Europe.** This is carried out for the 2050s (2040-59 average), left column (a, b, c, d) and the 2080s (2070-89 average), right column (e, f, g h), from the lowest (RCP2.6, top, a, e) to the highest (RCP8.5, bottom, d, h) emission scenario.


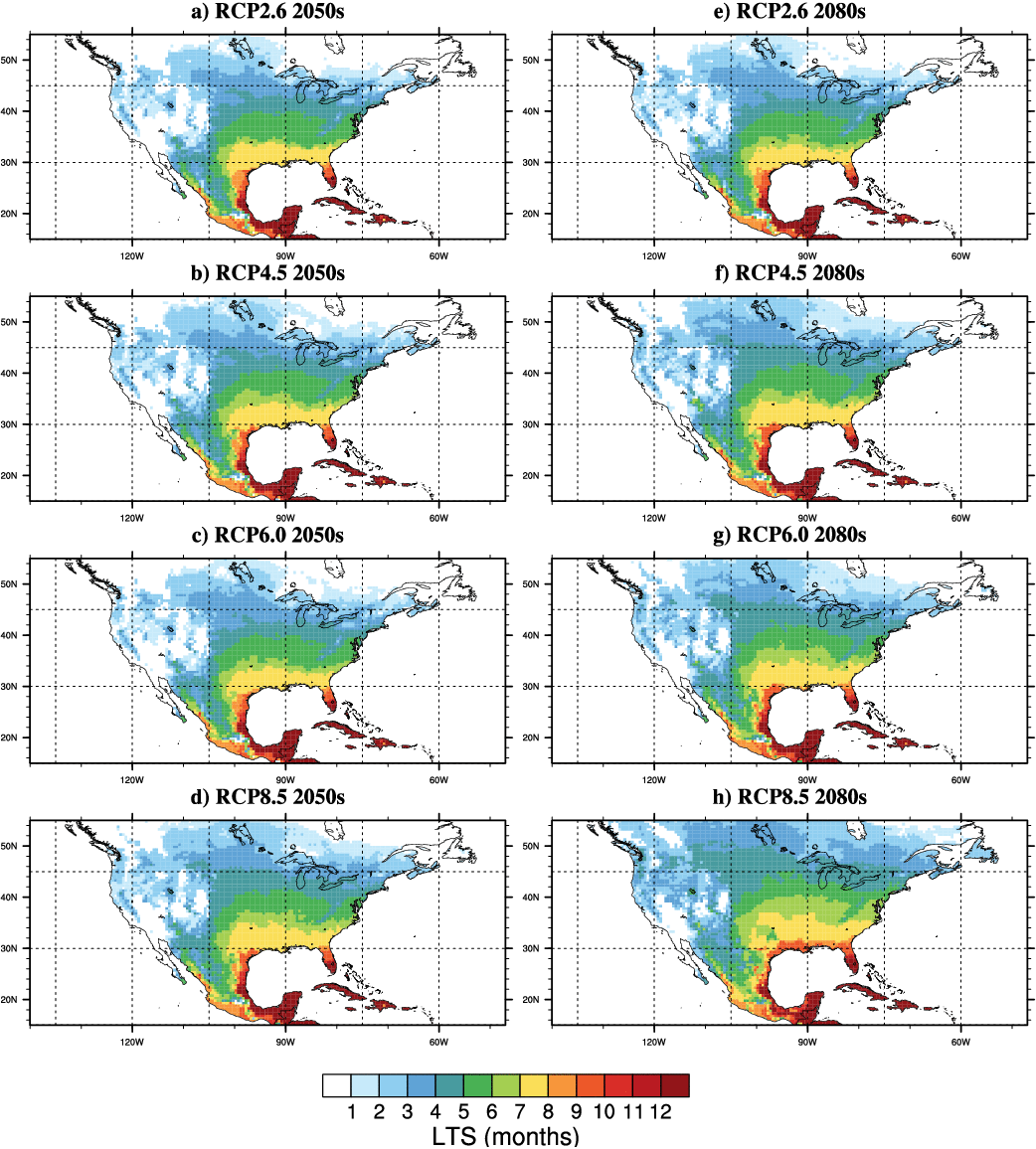


**Figure S9**: **Simulated length of the ZIKV transmission season by *Ae. albopictus* over North America.** This is carried out for the 2050s (2040-59 average), left column (a, b, c, d) and the 2080s (2070-89 average), right column (e, f, g h), from the lowest (RCP2.6, top, a, e) to the highest extreme (RCP8.5, bottom, d, h) emission scenario.


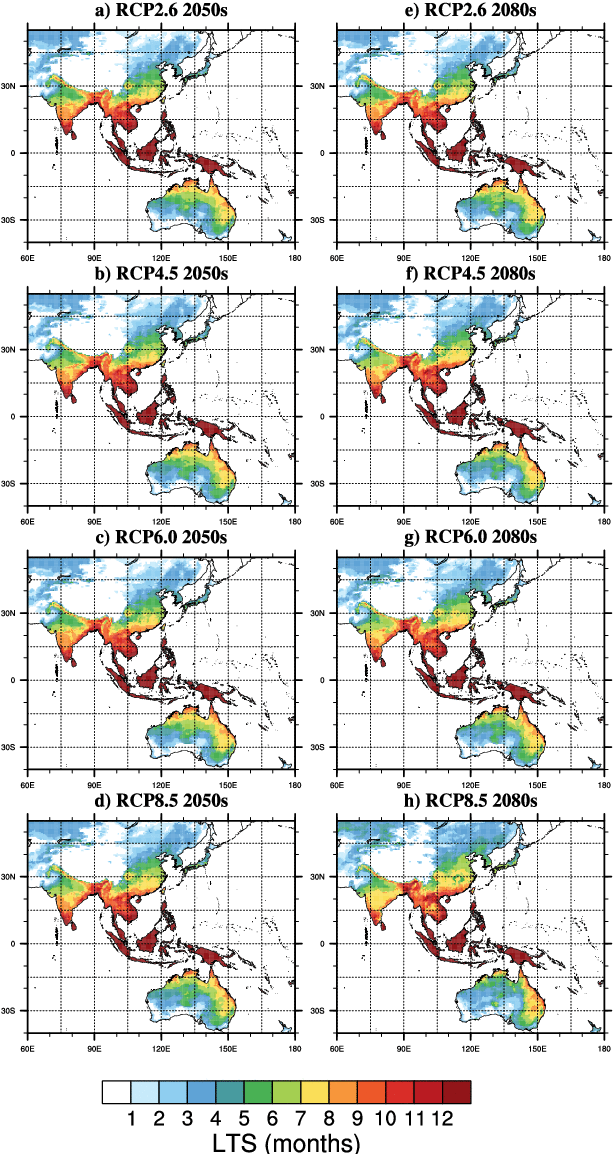


**Figure S10**: **Simulated length of the ZIKV transmission season by *Ae. albopictus* over Asia.** This is carried out for the 2050s (2040-59 average), left column (a, b, c, d) and the 2080s (2070-89 average), right column (e, f, g h), from the lowest (RCP2.6, top, a, e) to the highest (RCP8.5, bottom, d, h) emission scenario.


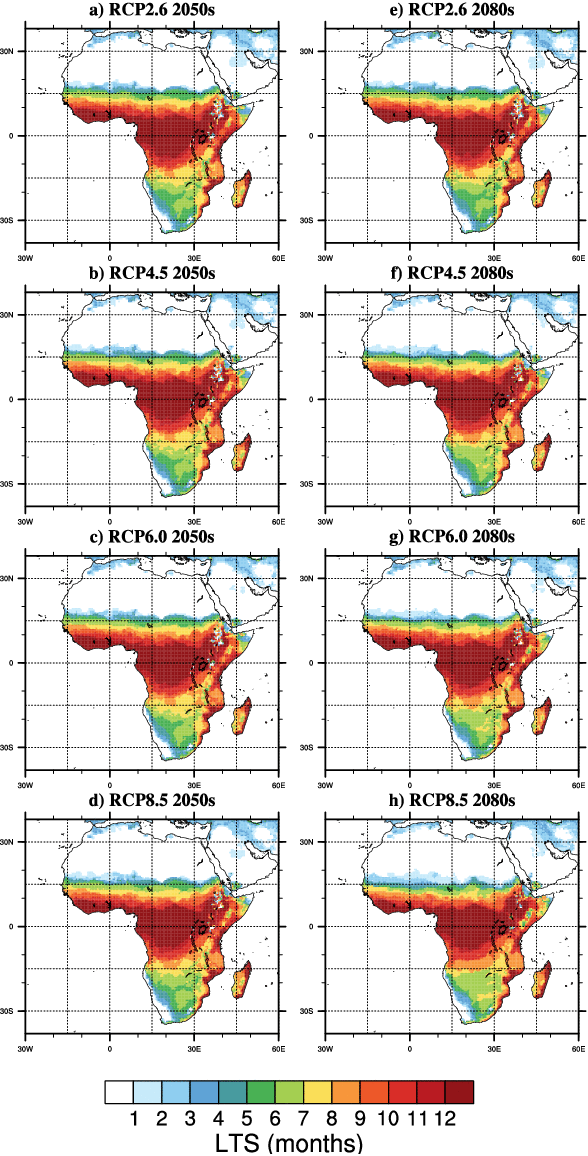


**Figure S11**: **Simulated length of the ZIKV transmission season by *Ae. albopictus* over Africa.** This is carried out for the 2050s (2040-59 average), left column (a, b, c, d) and the 2080s (2070-89 average), right column (e, f, g h), from the lowest (RCP2.6, top, a, e) to the highest (RCP8.5, bottom, d, h) emission scenario.


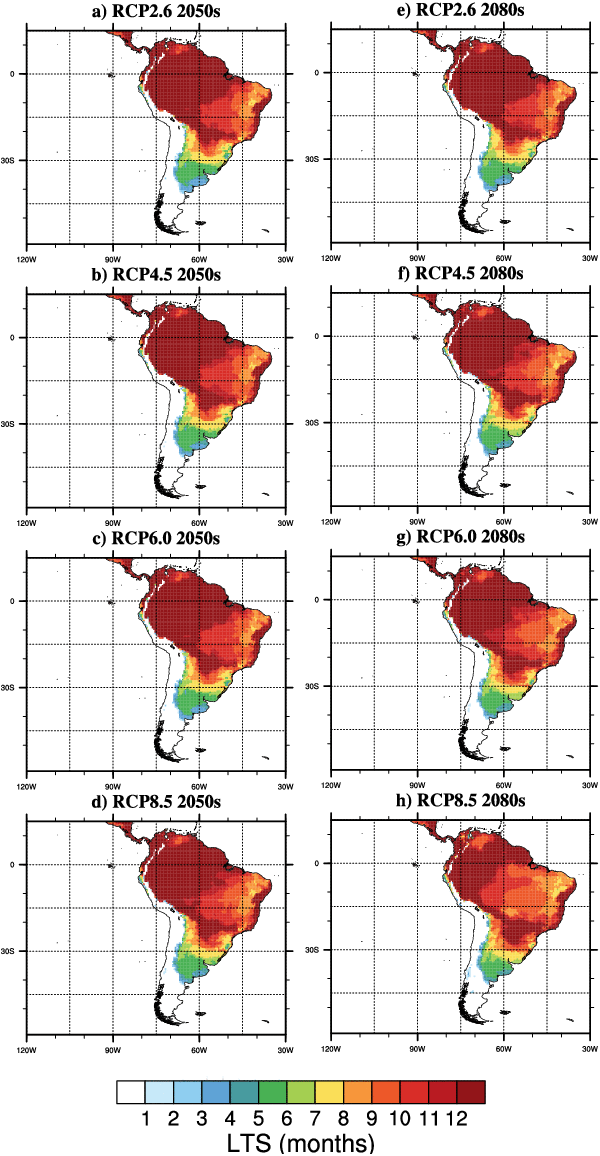


**Figure S12**: **Simulated length of the ZIKV transmission season by *Ae. albopictus* over South America.** This is carried out for the 2050s (2040-59 average), left column (a, b, c, d) and the 2080s (2070-89 average), right column (e, f, g h), from the lowest (RCP2.6, top, a, e) to the highest (RCP8.5, bottom, d, h) emission scenario.

**References**

1. Caminade C, Turner J, Metelmann S, Hesson JC, Blagrove MSC, Solomon T, Morse AP, Baylis M. 2017 Global risk model for vector-borne transmission of Zika virus reveals the role of El Niño 2015. *Proc. Natl. Acad. Sci.* **114**, 119–124. (doi:10.1073/pnas.1614303114)

2. Tesla B, Demakovsky LR, Mordecai EA, Ryan SJ, Bonds MH, Ngonghala CN, Brindley MA, Murdock CC. 2018 Temperature drives Zika virus transmission: evidence from empirical and mathematical models. *Proc. R. Soc. B Biol. Sci.* **285**, 20180795. (doi:10.1098/rspb.2018.0795)

3. Romeo-Aznar V, Paul R, Telle O, Pascual M. 2018 Mosquito-borne transmission in urban landscapes: the missing link between vector abundance and human density. *Proc. R. Soc. B Biol. Sci.* **285**, 20180826. (doi:10.1098/rspb.2018.0826)

4. Kraemer MU *et al.* 2015 The global distribution of the arbovirus vectors *Aedes aegypti* and *Ae. albopictus*. *Elife* **4**, e08347. (doi:10.7554/eLife.08347)

5. Craig MH, Snow RW, le Sueur D. 1999 A climate-based distribution model of malaria transmission in sub-Saharan Africa. *Parasitol. Today* **15**, 105–11.

6. Liu-Helmersson J, Stenlund H, Wilder-Smith A, Rocklöv J. 2014 Vectorial Capacity of Aedes aegypti: Effects of Temperature and Implications for Global Dengue Epidemic Potential. *PLoS One* **9**, e89783. (doi:10.1371/journal.pone.0089783)

7. Scott TW, Amerasinghe PH, Morrison AC, Lorenz LH, Clark GG, Strickman D, Kittayapong P, Edman JD. 2000 Longitudinal studies of Aedes aegypti (Diptera: Culicidae) in Thailand and Puerto Rico: blood feeding frequency. *J. Med. Entomol.* **37**, 89–101.

8. Guzzetta G, Poletti P, Montarsi F, Baldacchino F, Capelli G, Rizzoli A, Rosà R, Merler S. 2016 Assessing the potential risk of Zika virus epidemics in temperate areas with established *Aedes albopictus* populations. *Eurosurveillance* **21**, 30199. (doi:10.2807/1560-7917.ES.2016.21.15.30199)

9. Brady OJ *et al.* 2013 Modelling adult Aedes aegypti and Aedes albopictus survival at different temperatures in laboratory and field settings. *Parasit. Vectors* **6**, 351. (doi:10.1186/1756-3305-6-351)

10. Musso D, Roche C, Nhan T-X, Robin E, Teissier A, Cao-Lormeau V-M. 2015 Detection of Zika virus in saliva. *J. Clin. Virol.* **68**, 53–55. (doi:10.1016/j.jcv.2015.04.021)

11. Fan Y, van den Dool H. 2008 A global monthly land surface air temperature analysis for 1948–present. *J. Geophys. Res.* **113**, D01103. (doi:10.1029/2007JD008470)

12. Schneider U, Becker A, Finger P, Meyer-Christoffer A, Ziese M, Rudolf B. 2014 GPCC’s new land surface precipitation climatology based on quality-controlled in situ data and its role in quantifying the global water cycle. *Theor. Appl. Climatol.* **115**, 15–40. (doi:10.1007/s00704-013-0860-x)

13. WMO. 2017 *WMO Guidelines on the Calculation of Climate Normals*. Geneva: WMO.

14. Hempel S, Frieler K, Warszawski L, Schewe J, Piontek F. 2013 A trend-preserving bias correction &amp;ndash; the ISI-MIP approach. *Earth Syst. Dyn.* **4**, 219–236. (doi:10.5194/esd-4-219-2013)

15. R Core Team. 2018 R: a language and environment for statistical computing. *R Found. Stat. Comput. Vienna, Austria.* See https://www.r-project.org/ (accessed on 10 July 2019).

16. Caminade C, Turner J, Metelmann S, Hesson JC, Blagrove MSC, Solomon T, Morse AP, Baylis M. 2017 Global risk model for vector-borne transmission of Zika virus reveals the role of El Niño 2015. *Proc. Natl. Acad. Sci. U. S. A.* **114**, 119–124. (doi:10.1073/pnas.1614303114)
